# Effects of ivabradine on myocardial autophagia and apoptosis in isoprenaline-induced heart failure in mice

**DOI:** 10.22038/IJBMS.2023.70060.15236

**Published:** 2024

**Authors:** Menghua Sun, Feiya Yin, Xinrong Wu, Shaoer Sun, Yongqiang An, Manlin Zhu, Xiaomin Li, Wei Liu

**Affiliations:** 1 Department of Cardiology, Fourth Affiliated Hospital of Harbin Medical University, Harbin 150001, China; 2 The University of Sydney, Newtown, NSW 2042, Australia; 3 Department of Geriatric Cardiology, Guangdong Provincial People’s Hospital, Guangzhou, PR. China, 510080; # These authors contributed equally to this work

**Keywords:** Apoptosis, Autophagy, Cardiac remodeling, Fibrosis, Heart failure

## Abstract

**Objective(s):**

To investigate the effects and mechanisms of ivabradine (IVA) on isoprenaline-induced cardiac injury.

**Materials and Methods:**

Forty male C57BL/6 mice were randomly divided into control group, model group, high-dose IVA group, and low-dose IVA group. The control group was given saline, other groups were given subcutaneous injections of isoproterenol (ISO) 5 mg/kg/d to make the myocardial remodeling model. A corresponding dose of IVA (high dose 50 mg/kg/d, low dose 10 mg/kg/d) was given by gavage (30 days). A transthoracic echocardiogram was obtained to detect the structure and function of the heart. An electron microscope was used to explore the cardiomyocytes’ apoptosis and autophagy. HE staining and Masson’s trichrome staining were performed to explore myocardial hypertrophy and fibrosis. Western blot was used to detect Bax, Bcl-2, cleaved caspase-3, Becline-1, LC3, phosphorylated p38 mitogen-activated protein kinase (p-p38MAPK), phosphorylated extracellular regulated protein kinases1/2 (p-ERK1/2), phosphorylated c-Jun N-terminal kinase (p-JNK), and α-smooth muscle actin (α-SMA) in the myocardium.

**Results:**

Heart rate in the IVA groups was reduced, and the trend of heart rate reduction was more obvious in the high-dose group. Echocardiography showed that IVA improved the cardiac structure and function compared to the model group. IVA attenuated cardiac fibrosis, decreased cardiomyocyte apoptosis, and increased autophagy. The phosphorylated MAPK in the ISO-induced groups was increased. IVA treatment decreased the p-p38MAPK level. There were no differences in p-ERK and p-JNK levels.

**Conclusion:**

The beneficial effects of IVA on myocardial injury are related to blocking the p38MAPK signal pathway, decreasing cardiomyocyte apoptosis, and increasing cardiomyocyte autophagy.

## Introduction

Heart failure (HF) is a complex clinical syndrome with a high incidence worldwide (1, 2). It causes high morbidity and mortality and has a huge adverse impact on the quality of life (3). Although there are various types of drugs and devices available for HF treatment, the long-term prognosis is not ideal (4). Heart rate acceleration is a direct indicator of poor prognosis of HF (5). Ivabradine (IVA), a heart rate-slowing drug developed in recent years, can enter the hyperpolarized activated cyclic nucleotide-gated (HCN) channel and bind to the intracellular side to inhibit I*f *current (6). There are four subtypes of HCN channels (HCN1-4), which are not only expressed in the sinoatrial node and cardiac conduction system but also highly expressed in cardiac muscle and nerve tissue (7-9).

IVA is clinically used to treat chronic stable angina pectoris and HF (10,11). Clinical trials have shown that IVA can reduce angina symptoms in patients with stable coronary artery disease, but it cannot improve clinical outcomes (12). In contrast, in symptomatic HF patients, IVA significantly improved the clinical prognosis while alleviating symptoms (13). The underlying mechanisms of the different prognostic effects of IVA on angina and HF have not been elucidated. Studies suggest that in addition to slowing down heart rate, the beneficial effect of IVA on the prognosis of HF also comes from its pleiotropic effects, including reducing the formation of reactive oxygen species in the mitochondria of cardiomyocytes (14). It is speculated that the hyperpolarized activated cyclic nucleotide-gated channels in mitochondria are also targets of IVA (15). Therefore, IVA may improve the related functions of mitochondria in failing hearts, such as regulating apoptosis and autophagy, and thus may achieve additional protective effects in addition to slowing heart rate. Mitochondria function is closely related to the MAPK signaling pathway, including ERK2-ERK1, JNK, and p38MAPK, which are all involved in the regulation of autophagy and apoptosis (16, 17). 

Myocardial remodeling is a common pathophysiological process in which chronic sympathetic activation is considered to be one of the key factors in its occurrence and development. Isoproterenol (ISO) is a synthetic catecholamine, long-term low-dose ISO can induce myocardial fibrosis (18). In this study, we used ISO as an inducer of cardiac remodeling in mice and examined the effect and underlying mechanisms of IVA.

## Materials and Methods

***Experimental animals***

Forty 6-week-old male C57BL/6 mice, weighing 18-22 g, were provided by the Weitonglihua laboratory animal center (Beijing, China). The mice were randomly divided into a control group (n=10), model group (n=10), high-dose IVA group (n=10), and low-dose IVA group (n=10). The model group, high-dose IVA group, and low-dose IVA group were given subcutaneous ISO [Sigma-Aldrich (St. Louis, MO, USA)] (5 mg/kg/d) from day 1 to day 30. High-dose and low-dose IVA groups were given IVA (SERVIER, France) (high dose 50 mg/kg/d; low dose 10 mg/kg/d) for 30 days. Isoproterenol and IVA were given sequentially to every mouse per day. The control group was given the same amount of normal saline subcutaneous injection and gavage.

***Blood pressure and heart rate measurement***

The blood pressure and heart rate of all animals were measured on the first day of the experiment and before the end of the experiment. The heart rate, systolic blood pressure, diastolic blood pressure, and mean blood pressure of the mice were measured by using the Softron BP-2010 series (Softron Biotechnology Co., Ltd, Beijing, China). Indirect measurement of the mouse tail artery was performed while keeping the room temperature at about 30 °C in a quiet and awake state. We install the mouse into the sphygmomanometer pulse pressure sleeve and cover the pressure transducer at nearly 1/3 of the tail root of the mouse. At the same time, we put the mouse into the heater and set the temperature to 39 ^°^C. When the tail temperature of the mice rises, the skin turns pink, and the tail vein expands, pressure should be applied until the blood flow of the tail artery is blocked; continue to pressurize to 180-190 mmHg, then release evenly and slowly. The heart rate and blood pressure can be read out in the software. The above method is repeated 5 times, and 3-4 qualified cycle parameters selected to take the average value.

***Echocardiography***

The mice were sedated intraperitoneally with phenobarbital, then fixed in the supine position. The chest was shaved with scissors. A PHILIPS-EPIQ 5 transthoracic echocardiogram (Bothell, WA, USA) was obtained by experienced ultrasound doctors using an S12-4 MHz imaging linear scan probe transducer. Left ventricular posterior wall thickness at end-diastole (LVPWd), interventricular septum thickness at end-systole (IVSs), left ventricular diastolic diameter (LVIDd), interventricular septum thickness at end-diastole (IVSd), left ventricular posterior wall diameter at end-systole (LVPWs), left ventricular internal diameter in systole (LVIDs), and left ventricular ejection fraction (LVEF) were measured. The percentage of fractional shortening (FS) was then calculated.

***Transmission electron microscope***

Transmission electron microscopy was used to observe myocardial ultrastructure. After the heart was quickly harvested, a sharp scalpel was used to quickly cut the apical tissue of the left ventricle with a thickness of about 2 mm along the direction of the myofilament, and immediately put it into the precooled 2.5% glutaraldehyde for fixation to prepare it for transmission electron microscopy. After Uranium acetate and lead citrate double staining, transmission electron microscopy (Hitachi H-7650) was used to observe and a typical visual field was selected to take photos.

***Histopathological staining***

Apical tissue samples were dehydrated, embedded in paraffin, and cut into 5-μm thick sections. Hematoxylin-eosin (HE) staining was used to evaluate the pathological changes and myocardial injury. The average cross-sectional area (CSA) of 40-60 cardiomyocytes under the 200x microscope was calculated. Masson trichrome staining was used to assess the degree of collagen deposition. The area percentage was calculated by the ratio of collagen deposition area to the total visual area, using the ImageJ software.

***Western blot***

Myocardial tissue protein was extracted and protein concentration was determined by the BCA method. SDS-polyacrylamide gel electrophoresis was performed for 1.5 hr. The membrane was transferred and immersed in the sealing solution for incubation for 1 hr. 1:500 diluted primary antibodies [anti-p-p38MAPK, anti-p-ERK1/2, anti-p-JNK, anti-LC3, anti-Beclin1, anti-Bcl-2, anti-Bax, anti-Caspase3, and anti-α-SMA] were added and incubated at 4 ^°^C overnight. The second antibody was incubated at room temperature for 45 min. The film was scanned and the optical density of the target strip analyzed using a Gel image processing system (gEL-Pro-Analyzer software). β-actin was used as the internal reference.

***Statistical analysis***

SPSS 20.0 statistical analysis software was used, and the experimental data were expressed as mean±standard deviation. One-way analysis of variance (ANOVA) was conducted with Bonferroni *post hoc* analysis to examine the differences between groups, and *P*<0.05 was considered statistically significant.

## Results

***Blood pressure and heart rate***

Before the experiment, there were no differences in blood pressure and heart rate among any groups (*P*>0.05). Heart rate was increased in ISO-induced model groups compared to the control group (*P*<0.05). Compared with the model group, the heart rate of low-dose and high-dose IVA groups was decreased (*P*<0.05). The trend of heart rate reduction was more obvious in the high-dose IVA group. There was no difference in blood pressure among any groups (*P*>0.05) (Table 1).

***Cardiac structure and function***

**Figure 1:**
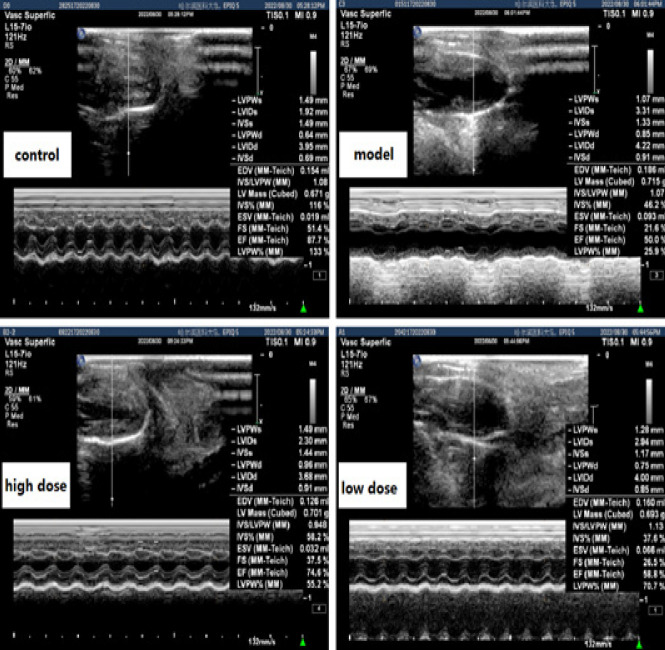
Typical echocardiography results of mice in the control, model, high dose and low dose IVA group

The cardiac function of mice was measured by echocardiography. In the model group, there was an increase in LVIDd and LVIDs, as well as a decrease in LVEF and FS compared to the control group (*P<*0.05). Low dose and high dose IVA treatment improved the cardiac function, as indicated by increasing the LVEF and FS index compared to the model group (*P<*0.05). The indicator of heart size, LVIDd and LVIDs, were also alleviated by IVA treatment (*P<*0.05). There was no significant differences in IVSd, IVSs, LVPWd and LVPWs among the four groups (*P>*0.05) (Table 2, Figure 1).

***Histopathology results***

In the control group, HE staining showed normal morphology of cardiomyocytes, orderly arrangement, unbroken muscle fibers, and clear nucleus. However, in the model group, cardiomyocytes were disturbed, inflammatory cells infiltrated, cardiomyocytes hypertrophy, and myocardial fiber breakage showed wavy changes. As HE-stained heart sections showed, compared with the control group, the mice in the ISO group showed cardiac hypertrophy, which was alleviated after IVA treatment (Figure 2-1 A-D). The CSA of cardiomyocytes was higher in the ISO groups than in the control group (*P<*0.05). IVA therapy decreased the CSA index in the high dose and low dose groups compared with the model group (*P<*0.05).

As shown in Masson trichrome staining, there was extensive collagen deposition in the heart of the model group compared with the control group, whereas treatment with IVA greatly reduced contents of collagen deposition. Compared with the model group, fibrosis affected areas were reduced in the low-dose and high-dose IVA groups (*P<*0.05). There was no difference in the degree of fibrosis between the low-dose IVA group and the high-dose IVA group (Figure 2-2 I-L).

***Apoptosis and autophagy in the myocardium***

Transmission electron microscopy showed that the cardiomyocytes in the control group had uniform morphology, normal structure, and normal chromatin, and occasionally apoptosis and autophagy were found. In the model group, myocardial cells showed apoptosis such as cytoplasmic separation, nuclear chromatin concentration, edge aggregation, and increased density, as well as typical apoptotic bodies, nuclear hypertrophy, and malformation. The number of autophagosomes with organelles, proteins, and other substances wrapped in double membranes in the high-dose and low-dose IVA groups was higher than that in the model group, and apoptosis was lower than that in the model group (Figure 3-1).

Western blot analysis showed that compared with the model group, anti-apoptotic protein Bcl-2 was increased in the high-dose and low-dose IVA groups (*P*<0.05), pro-apoptotic protein Bax and cleaved-capase-3 were decreased (*P*<0.05). Autophagy-related protein LC3 and Beclin-1 were increased (*P*<0.05) in the IVA groups compared to the model group (Figure 3-2).

***Phosphorylated MAPK expression***

The phosphorylated MAPK protein in the ISO-induced groups was increased (*P*<0.05), and IVA treatment decreased p-p38MAPK level (*P*<0.05). There were no differences in p-ERK and p-JNK protein levels (Figure 4).

***Expression of α-SMA in the myocardium***

Western blot results indicated that the expression of α-SMA in the model group was higher than that in the control group (*P*<0.05). The expression levels were decreased in the two IVA groups compared with the model group (*P*<0.05)(Figure 4).

## Discussion

Cardiomyocyte apoptosis is cell death caused by the initiation of specific programs under complex regulatory mechanisms and is an important mechanism for the transition from myocardial hypertrophy to HF (19, 20). Bcl-2 protein can control the activation of downstream caspase proteases, which are key proteins in the mitochondrial apoptosis pathway (21, 22). After activating the caspase family, proteases undergo cleavage, and cleaved caspase-3 is the “executor of apoptosis” (23). In the present study, ISO led to decreased expression of Bcl-2 and increased expression of Bax and cleaved caspase-3 in cardiac tissue, and apoptotic cardiomyocytes were observed by electron microscopy. We found that different doses of IVA increased the expression of Bcl-2, decreased the expression of Bax and cleaved caspase-3 protein, and inhibited cardiomyocyte apoptosis. IVA inhibits cardiomyocyte apoptosis by regulating endogenous apoptotic pathways, up-regulating anti-apoptotic protein, and inhibiting pro-apoptotic protein expression.

Our study confirmed the existence of cardiac remodeling in mice with ISO-induced HF by HE staining, Masson trichrome staining, and cardiac ultrasound. In the process of cardiac remodeling, autophagy on the one hand inhibits the accumulation of abnormal proteins and damaged organelles that damage cardiac function, and on the other hand ensures the energy demand of cardiomyocytes (24). Studies have found that the process of cardiac hypertrophy is accompanied by a decrease in autophagy level, and blocking the autophagy of cardiomyocytes can promote the process of cardiac remodeling (25). In the present study, the LC3 protein expression increased after subcutaneous injection of ISO for 30 days, which may be the self-regulation of the myocardium under the action of the sympathetic nerve and the increase of energy supply through the up-regulation of autophagy, which is consistent with the previous findings of Nakai *et al*. (24-26). Different doses of IVA promoted the expression of Beclin-1, LC3 proteins and increased the level of cardiac autophagy. We conclude that the regulation of autophagy by IVA is one of the possible mechanisms of IVA against ISO-induced myocardial injury.

MAPK signaling pathway can transmit signals generated by various extracellular stimuli from the cell membrane to the nucleus and participate in the process of apoptosis and autophagy (27). In this study, we found that p-p38MAPK, p-ERK1/2, and p-JNK in the myocardium of the model group increased, but after IVA treatment, the other two phosphorylases did not decrease, while the expression of p38MAPK decreased. It is speculated that the mechanism of IVA on myocardial remodeling may be related to inhibiting the activation of phosphorylated p38MAPK, thus further regulating myocardial apoptosis and autophagy (28).

In the normal heart, an increase in heart rate leads to an increase in systolic function, which is called the Treppe effect or the Bowditch effect (29). The Treppe phenomenon reflects improved excitation-contraction coupling dynamics. However, in the failing heart, the Treppe effect is the opposite: systolic function decreases with increasing heart rate (30). This may also be part of the reason that IVA improves the prognosis of patients with HF but does not improve the prognosis of patients with angina pectoris. Decreased heart rate not only prolonged myocardial diastole, and increased blood flow and oxygen supply, but also decreased myocardial oxygen demand and increased myocardial oxygen supply-demand ratio (31), which may also be a factor to improve HF. According to the SHIFT trial with systolic HF patients, the magnitude of heart rate reduction with IVA depended on the baseline heart rate (32). In fact, the Treppe phenomenon also varies from species to species, and the heart rate of mice is much higher than that of humans.

**Table 1:** Blood pressure and heart rate in the control, model, high dose IVA and low dose IVA group

	n	HR		SBP		MBP		DBP	
		before	after	before	after	before	after	before	after
control	10	540±46	527±36	103±13	102±13	82±9	79±14	70±8	67±15
model	10	566±35	602±34^＃^	103±17	113±17	79±11	84±11	67±11	69±11
High dose IVA	10	560±32	362±23*^&^	102±17	110±13	78±11	86±13	67±11	75±12
Low dose IVA	10	565±14	449±21*	106±15	110±13	78±7	84±5	63±6	71.5±3

Data were expressed as mean±standard deviation, # : compared with the control group, *P<*0.05, * : compared with the model group, *P<*0.05, & : compared with the low-dose group, *P<*0.05

HR: heart rate, SBP: systolic blood pressure, MBP: mean blood pressure, DBP: diastolic blood pressure. Before: before the experiment; after: after the experiment

**Table 2:** Echocardiographic parameters in the four groups

	control	model	high dose IVA	low dose IVA
LVIDd (mm)	4.0±0.1	4.16±0.8	3.9±0.7	4.14±0.7
LVIDs (mm)	2.08±0.5	3.2±0.1	2.35±0.2	2.89±0.1
IVSd (mm)	0.69±0	0.91±0#	0.82±0.1	0.78±0.1
IVSs (mm)	1.51±0.2	1.35±0.2	1.24±0.2	1.21±0.1
LVPWd (mm)	0.67±0.3	0.87±0.2	0.82±0.1	0.75±0
LVPWs (mm)	1.42±0.1	1.1±0.1	1.48±0.1	1.28±0.1
LVEF (%)	84.9±5.5	52.0±4.7 #	76.4±5.7 *	58.8±2.6 #*
FS (%)	48.1±0.4	22.7±0.2#	39.7±0.2*	26.5±0.3#	

^#^ *P<*0.05 versus control group. ^*^ *P<*0.05 versus model group

LVIDd: left ventricular diastolic diameter; LVIDs: left ventricular internal diameter in systole; IVSd: interventricular septum thickness at end-diastole; IVSs: interventricular septum thickness at end-systole; LVPWd: left ventricular posterior wall thickness at end-diastole; LVPWs: left ventricular posterior wall diameter at end-systole; LVEF: left ventricular ejection fraction; FS: fractional shortening

C57BL/6 mice were divided into control group (n=10), model group (n=10), high-dose IVA group (n=10), and low-dose IVA group (n=10). Model group, high-dose IVA group, and low-dose IVA group were given subcutaneous ISO (5 mg/kg/d) from day 1 to day 30. High-dose (50 mg/kg/d) and low-dose (10 mg/kg/d) IVA groups were treated with IVA for 30 days. In echocardiography examination, LVPWd, IVSs, LVIDd, IVSd, LVPWs, LVIDs, and LVEF were measured. Percent of FS was then calculated.

LVIDd: left ventricular diastolic diameter; LVIDs: left ventricular internal diameter in systole; IVSd: interventricular septum thickness at end-diastole; IVSs: interventricular septum thickness at end-systole; LVPWd: left ventricular posterior wall thickness at end-diastole; LVPWs: left ventricular posterior wall diameter at end-systole; LVEF: left ventricular ejection fraction; FS: fractional shortening

**Figure 2-1:**
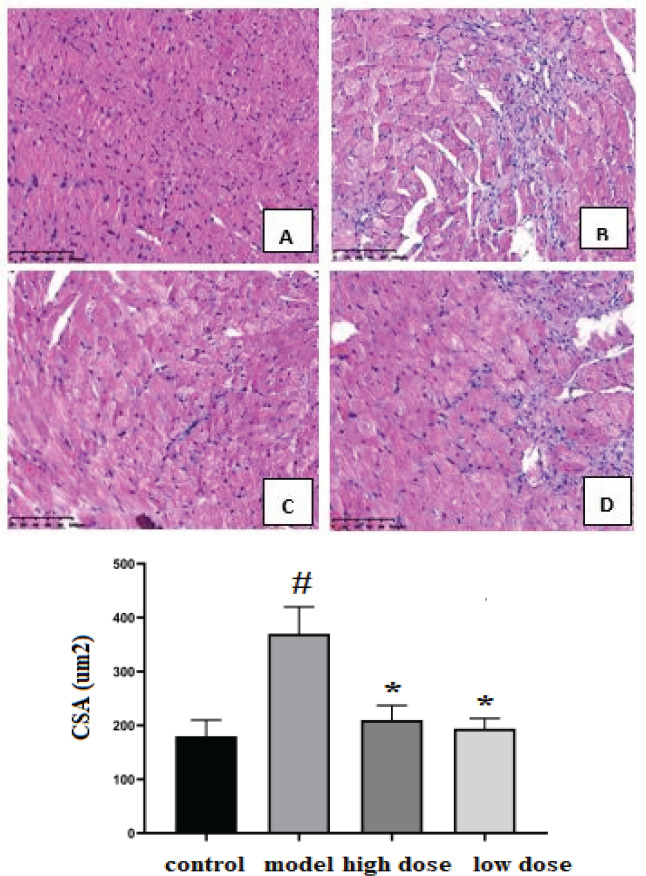
Hematoxylin-eosin (HE) staining (cross-section) and the cross-sectional area (CSA) results in each group

A: control group; B: model group; C: IVA high dose group; D: IVA low dose group.

#: Compared with the control group, *P<*0.05; *: Compared with the model group, *P<*0.05

**Figure 2-2:**
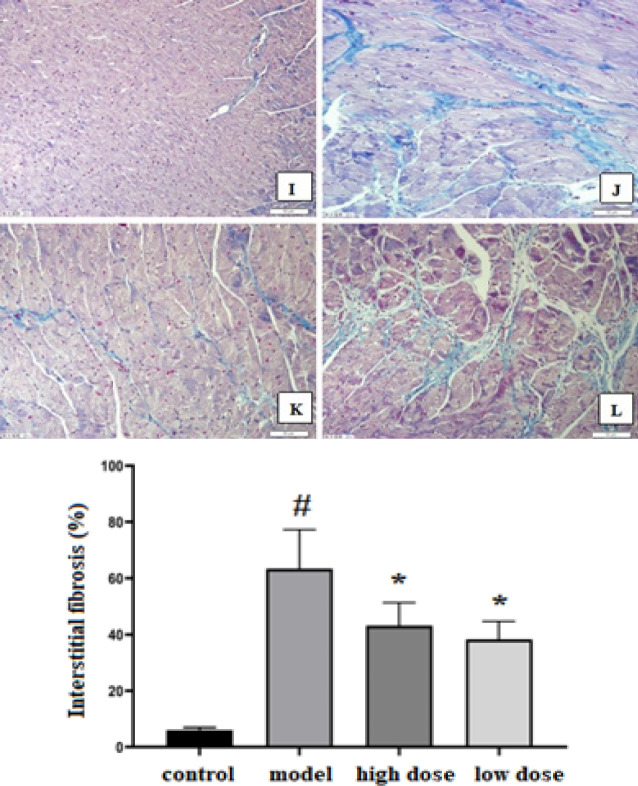
Masson trichrome staining results in each group

I: control group; J: model group; K: IVA high dose group; L: IVA low dose group. #: Compared with control group, *P<*0.05; *: Compared with model group, *P<*0.05

**Figure 3-1:**
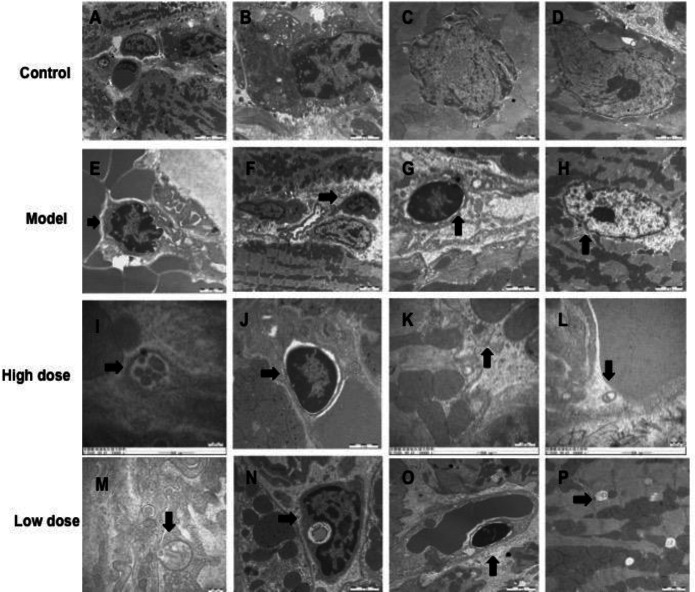
Transmission electron microscope scanning results of cardiomyocytes in each group

(A)~(D): normal myocardial cell structure; (E) apoptotic bodies; (F), (G): the cells are undergoing apoptosis; (H) hypertrophic cardiomyocytes; (I)~(P): autophagic cardiomyocytes; (I): autophagosome. Scale=2 μm

**Figure 3-2:**
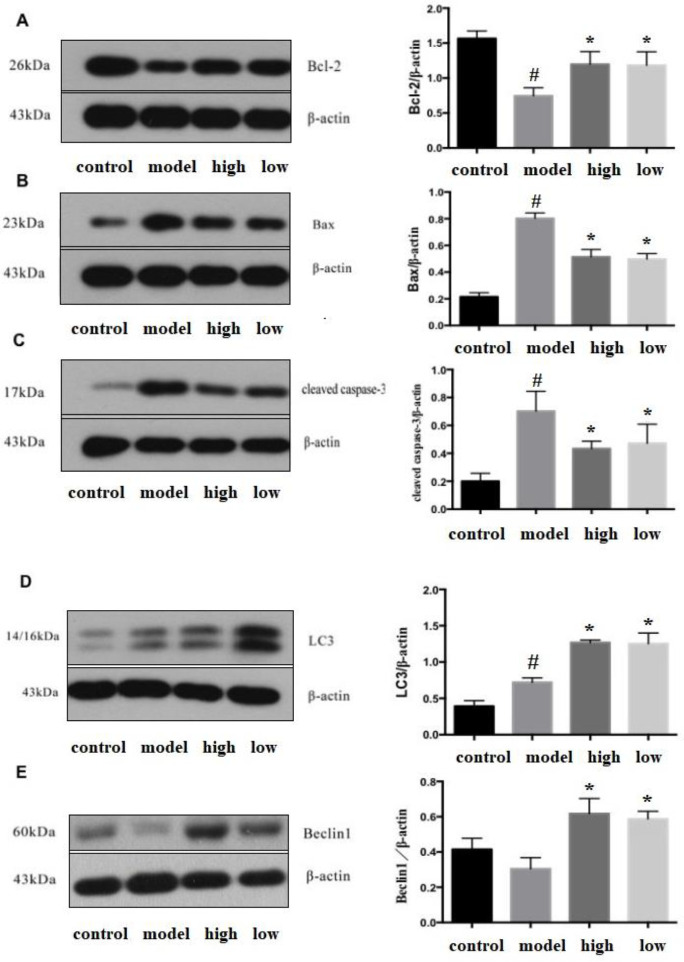
Apoptosis and autophagy-related protein expression

#: Compared with control group, *P<*0.05; *: Compared with model group, *P<*0.05

Bcl-2: B-cell lymphoma-2; BAX: Bcl-2-associated X; LC3: microtubule-associated protein light chain 3

**Figure 4:**
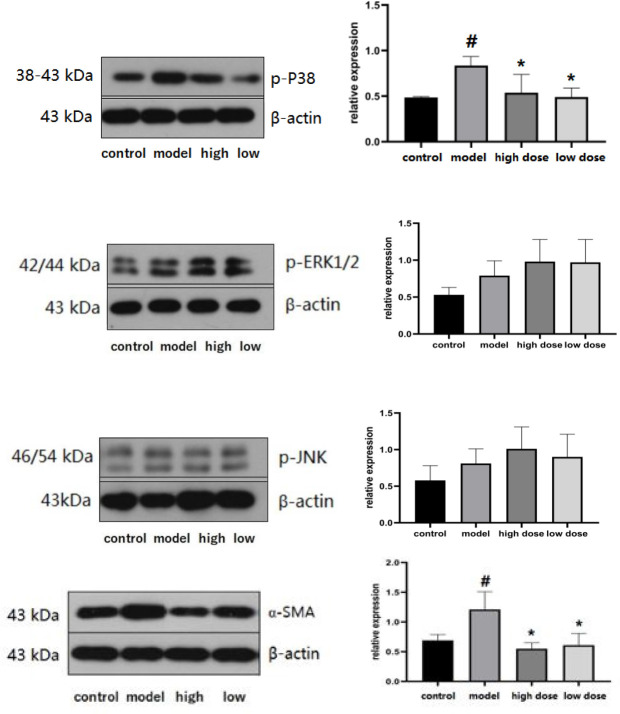
Phosphorylated MAPK in myocardial tissues in each group was detected by Western blot

#: Compared with control group, *P<*0.05; *: Compared with model group, *P<*0.05

P-p38: Phosphorylated p38 mitogen-activated protein kinase; α-SMA: α-smooth muscle actin

## Conclusion

Our study confirmed in the mice model of ISO-induced HF that the beneficial effect of IVA on myocardial remodeling is related to blocking the p38MAPK signaling pathway, reducing cardiomyocyte apoptosis, and increasing cardiomyocyte autophagy. This study will provide a theoretical basis for the expansion of the molecular mechanism of IVA and HCN channels in HF. The defect of this study is that the direct effect of IVA on the I*f* current was not detected in the *in vitro* experiments. In future studies, we will further design *in vitro* experiments to clarify the underlying association between HCN channels and p38MAPK signals.

## Authors’ Contributions

M S, F Y, and X W performed most of the experiments, data analysis, and statistical analysis and wrote the initial manuscript. S S, Y A, M Z, and X L performed the western blot experiment. W L designed the project, supervised the study, and analyzed the data.

## Conflicts of Interest

The writers declare no conflicts of interest associated with the present manuscript. 
